# Papain-like cysteine proteases in *Nicotiana benthamiana*: gene family members and their potential implications in recombinant protein expression

**DOI:** 10.3389/fpls.2025.1565487

**Published:** 2025-06-19

**Authors:** Yalun Yang, Zhichao Deng, Zhongqi Zhang, Yong Yang, Xiaolu Pan, Rongrong Wu, Tao Liu, Xiaoming Gao, Lingyan Li, Yongfeng Guo

**Affiliations:** ^1^ College of Agronomy, Qingdao Agricultural University, Qingdao, China; ^2^ Tobacco Research Institute, Chinese Academy of Agricultural Sciences, Qingdao, China; ^3^ Qingdao Municipal Key Laboratory of Plant Molecular Pharming, Tobacco Research Institute, Chinese Academy of Agricultural Sciences, Qingdao, China; ^4^ Vegetable and Landscape Gardening Institute, Heze Academy of Agricultural Sciences, Heze, China

**Keywords:** PLCP, *Nicotiana benthamiana*, biological stress, family analysis, gene expression patterns

## Abstract

**Introduction:**

Papain-like cysteine proteases (PLCPs), characterized by a conserved cysteine residue at their active sites, play crucial roles in plant growth, development, and responses to biotic and abiotic stresses. While their importance in plants is well recognized, the characterization of the PLCP gene family in *Nicotiana benthamiana* (*NbPLCP*) and its involvement in biotic stress responses remains insufficiently studied. This study aims to identify *NbPLCP* genes in *N. benthamiana* and elucidate their roles in facilitating heterologous protein expression.

**Methods:**

A comprehensive bioinformatics approach was employed to identify *NbPLCP* genes using *N. benthamiana* genomic data and homology with *Arabidopsis thaliana*. The analysis included examination of physicochemical properties, phylogenetic relationships, gene structures, conserved motifs, expression profiles, gene duplication events, and chromosomal distribution. Virus-induced gene silencing (VIGS) was utilized to screen for genes affecting heterologous protein expression in *N. benthamian*a.

**Results:**

We identified and characterized 50 *NbPLCP* members. Phylogenetic analysis classified these *NbPLCPs* into nine subfamilies, with uneven distribution across 19 chromosomes. Comparative analysis revealed closer evolutionary relationships between *N. benthamiana* and *Solanum lycopersicum*, followed by *Arabidopsis thaliana*, while showing more distant relationships with *Oryza sativa*. Functional studies demonstrated that *NbPLCPs* likely participate in regulating plant growth, development, and biotic stress responses. Importantly, VIGS-mediated silencing of *NbXCP1*, *NbXCP2*, and *NbXCP3* significantly enhanced the expression of heterologous GFP protein.

**Discussion:**

This study provides comprehensive insights into the PLCP family in *N. benthamiana*, highlighting their functional significance and potential in heterologous protein expression. Our findings establish a foundation for understanding the evolution and function of *NbPLCPs*, while demonstrating their potential applications in plant biotechnology for enhancing disease resistance and improving recombinant protein production systems. These results underscore the importance of *PLCPs* in both plant physiology and biotechnological applications.

## 1 Introduction

Proteases degrade substrate proteins by hydrolyzing peptide bonds. Based on the chemical nature of their catalytic sites, they can be classified into four categories: cysteine proteases, serine proteases, aspartic proteases, and metallo proteases (Zhang et al., 2023). Among them, cysteine proteases (CPs) rely on the cysteine residue at the active site as a nucleophilic group to catalyze reactions, a key subclass, papain-like cysteine proteases (PLCPs), is characterized by a conserved catalytic triad (Cys-His-Asn) at its core (Cen et al., 2019), by efficiently catalyzing reactions, they regulate plant growth and development, stress responses, and disease resistance processes (Liu et al., 2018; Li et al., 2025; Misas-Villamil et al., 2016; Rawlings et al., 2016). PLCPs are synthesized in the form of zymogens, with their precursors containing an N-terminal signal peptide, an autoinhibitory pro-domain, and a mature 25–35 kDa active protease domain. Some PLCPs also possess an unknown functional particle structure at the C-terminal (Zhang et al., 2023). These subfamilies include the CTB, ALP, RD19, SAG12, RD21, CEP, THI, XBCP, and XCP subfamilies (Kordis and Turk, 2009; Richau et al., 2012), Each subfamily achieves functional differentiation through specific structural domains, such as inhibitor-binding domains or substrate recognition regions (Long et al., 2025).

The activity of *PLCPs* is notably increased during the development and germination of seeds (Martinez et al., 2009), as well as in fruits (Lin et al., 1993) and various plant organs (van Wyk et al., 2014). In *Arabidopsis thaliana*, the gene *AtSAG12* exhibits a tightly regulated expression pattern associated with leaf senescence, and it has become a widely used molecular marker for studying leaf aging. Moreover, *AtRD21* and *AtRD19* serve as early-response marker genes for dehydration stress under drought and salt conditions, reflecting the role of PLCPs in the plant’s response to abiotic stresses (Guo and Gan, 2014; Lohman et al., 1994). In *Oryza sativa*, PLCPs assist in pathogen defense by broadly activating signaling pathways and transcription factors that coordinate downstream responses. This includes the upregulation of various disease-related proteins and the biosynthesis of secondary metabolites, which contribute to resistance mechanisms (Nino et al., 2020). As a critical component of the proteolytic machinery, PLCPs are responsible for the degradation of intracellular proteins, playing an essential role in regulating programmed cell death (PCD). PCD is a highly regulated biological process that is integral to numerous aspects of plant development, as well as responses to both biotic and abiotic stressors (Liu et al., 2018). In the context of *Medicago truncatula*, *MtCP6* is induced during both developmental and stress-induced nodule senescence. Its early expression promotes nodule senescence, while *MtCP77* positively regulates root nodule aging by accelerating plant PCD and reactive oxygen species (ROS) accumulation (Deng et al., 2019; Pierre et al., 2014). Furthermore, PLCPs are closely associated with plant resistance to herbivory. For instance, papain, a well-known PLCP found in the latex exudate of papaya, plays a critical role in defending the plant against herbivorous insects, such as lepidopteran larvae (Konno et al., 2004). This highlights the multifaceted role of PLCPs in both plant development and stress response, emphasizing their importance in the regulation of cellular processes and interactions with environmental factors. Although the functional roles of PLCPs have been extensively investigated in other species, research on this family in *N. benthamiana* remains unexplored.

Tobacco (*Nicotiana benthamiana*), a model plant, is widely utilized in the study of plant innate immunity and defense signaling pathways. It is particularly suitable for virus-induced gene silencing and transient gene overexpression through Agrobacterium-mediated infiltration (Bally et al., 2018). In recent years, *N. benthamiana* has gained attention as a biological reactor for the production of vaccines and therapeutic proteins (Douglas, 2018). However, the expression of exogenous proteins in *N. benthamiana* can be influenced by a variety of factors. One such factor is the interference by endogenous proteases in *N. benthamiana*, which can reduce the accumulation of exogenous proteins (Jutras et al., 2020). Among the various protease inhibitors identified, the co-expression of four specific inhibitors, namely SICYS8, NbPR4, NbPot1, and human HsTIMP, has been shown to significantly enhance the expression levels of exogenous proteins when compared to controls (Grosse-Holz et al., 2018).The SICYS8 protease inhibitor targets papain-like cysteine proteases. Strong inhibition of nine distinct PLCPs in *N. benthamiana* leaves has been observed upon the expression of SICYS8, suggesting a direct and potent effect on PLCP activity (Jutras et al., 2019). Although several members of the PLCP family have been extensively studied in *N. benthamiana*, a comprehensive investigation into the phylogeny of the entire PLCP gene family has yet to be undertaken.

In this study, we utilized the latest genomic data to systematically identify and analyze the PLCP gene family in *N. benthamiana*. Our comprehensive analysis included the construction of a phylogenetic tree, investigation of gene structures, identification of cis-regulatory elements, synteny analysis, expression profiling, and assessment of how specific PLCP family members influence the expression of the exogenous green fluorescent protein (GFP). These findings provide valuable insights into the evolutionary dynamics and functional roles of the PLCP gene family in *N. benthamiana*. Furthermore, this research contributes to a deeper understanding of *N. benthamiana* as a plant-based bioreactor, with potential applications in optimizing protein production within this species.

## 2 Materials and methods

### 2.1 Plant materials and growth conditions

The experimental materials used in this study were the *N. benthamiana* seeds kept by the Chinese Academy of Agricultural Sciences, and the designated tobacco seeds were sown on the soil matrix. After germination, the seedlings were transplanted to 25°C, 16 h/8 h (light/dark) photoperiod conditions to 6 to 8 leaf stage for Agrobacterium infiltration and inoculation.Samples were taken at 0h, 3 h, 12 h, 24 h, 36 h, 48 h and 72 h, and stored at -80°C after snap freezing in liquid nitrogen.

### 2.2 Identification and sequence analysis of the *NbPLCP* gene family

In order to search the members of the *PLCP* gene family, we downloaded the protein sequences of all the *Arabidopsis PLCP* family members from the TAIR (www.arabidopsis.org) *Arabidopsis* database, and analyzed these *PLCP* family members from the Pfam (http://pfam.xfam.org/) database, which showed that they generally possess the conserved domain of PF00112. Also we downloaded the genome and protein files of the NbeHZ1 version from the Nicomics (http://lifenglab.hzau.edu.cn/Nicomics/index.php) website. The hidden horse model file of PLCP gene family was found and downloaded through Pfam (http://pfam.xfam.org/) website using the hmmsearch program to set the E value of 1e-20 threshold; and the protein sequence of *AtPLCPs* family was Query to search the possible *NbPLCPs* sequence through BLAST search. The protein sequences obtained from both methods were removed after redundancy and uploaded to CDD (https://www.ncbi.nlm.nih.gov/Structure/bwrpsb/bwrpsb.cgi) and SMART (http://smart.embl.de/smart/set_mode.cgi?NORMAL=1) for domain validation to remove protein sequences without the PF000112 domain (Finn et al., 2014; Schultz et al., 1998).

By visiting the Expasy online platform (http://www.expasy.org/tools/protparam.html) and using its ProtParam tool, various physiochemical properties of NbPLCP protein members, including protein length, molecular weight, isoelectric point, aliphatic amino acid content and hydrophobicity index. The NbPLCP was named based on its chromosomal location (Gasteiger et al., 2003).

### 2.3 The evolutionary tree analysis of the *NbPLCPs* gene family members

The PLCP protein sequences of *Arabidopsis thaliana*, rice (*Oryza sativa*) and tomato (*Solanum lycopersicum*) can be downloaded from the Uniprot database (https://www.uniprot.org). A phylogenetic tree of these species and the PLCP proteins was constructed using MEGA11. Sequence ratios were performed using the ClustalW method, excluding non-conserved regions outside the aligned domains. A phylogenetic tree was constructed using a maximum likelihood method with a bootstrap value of 1000 (Kumar et al., 2018).

### 2.4 Analysis of gene structure, protein domains, and conserved motifs

Online tool MEME (http://meme-suite.org/) was used to predict conserved motifs in NbPLCP proteins, with motif lengths set to 6–100 with up to 10 motifs identified while maintaining default values for other parameters (Bailey et al., 2015). Using Batch CD-Search (https://www.ncbi.nlm.nih.gov/Structure/bwrpsb/bwrpsb.cgi) for the NbPLCP family, And the visualization of their protein-conserved domains using TBtools, Gene structure analysis based on the genomic DNA and CDS sequences of the NbPLCP gene family members, And using the online gene structure visualization server 2.0 (https://gsds.gao-lab.org/Gsds_help.php) for gene structure visualization.

### 2.5 Identification of cis-acting regulatory elements in the *PLCP* gene

From the 2000 bp region from the genome sequence file, the cis-regulatory elements (CAREs) of these promoter regions were then analyzed using PlantCARE software (http://bioinformatics.psb.ugent.be/webtools/plantcare/html/) (Lescot et al., 2002). Finally, tbtools presents all the identified elements of the promoter region of the *NbPLCP* gene as a heatmap.

### 2.6 Chromosomal localization and collinearity analysis

Information on the start and stop positions of *PLCPs* genes was extracted from the NbeHZ1 genome Gff3 file in TBtools software and mapped on the corresponding chromosome. For *NbPLCPs* analysis within species, TBtools software Fasta Tools and Blast tools, including chromosome length, gene location files and corresponding alignment files were used (Chen et al., 2020). *PLCPs* were analyzed using the One step MCScan X tool, and E value was set to 1e-5. The resulting Collinearity file was used for fragment duplication gene analysis and Tandem file was used for tandem repeat gene analysis (Wang et al., 2012). Use the KaKs_Calculator 3.0 software to calculate the non-synonymous to synonymous substitution ratio (Ka/Ks) of homologous gene pairs within the *NbPLCP* gene family (Zhang, 2022).

### 2.7 Experimental processing and sampling

Synthesize the nucleic acid sequence of SICYS8 (Sainsbury et al., 2013) and construct the *pEAQ::SCYS8* overexpression vector using T4 ligation technology. Inject *pEAQ::SCYS8* and *TRBO::GFP* into one side of the leaves of 4-week-old *Nicotiana benthamiana*, and co-inject empty pEAQ vector and *TRBO::GFP* into the other side of the leaves as a control group. Take photos and collect samples 3 days later, extract total protein from the samples, and use it for subsequent Western Blot analysis to detect the expression level of green fluorescent protein (GFP).

Design transient silencing fragments of the NbRD21, NbCTB, NbRD19, NbXBCP, and NbCEP subfamilies using the VIGS TOOL, Based on the conservative sequences of the same subfamily members, several vectors were designed to simultaneously silence multiple genes the primers used are shown in **Supplementary Table S1**. The specific vectors include: *TRV2::NbRD21BD* (silencing *NbRD21B* and *NbRD21D*), *TRV2::NbRD21E* (silencing *NbRD21E*), *TRV2::NbXBCP17 (silencing NbXBCP1 and NbXBCP7*), *TRV2::NbXBCP345* (silencing *NbXBCP3*, *NbXBCP4*, and *NbXBCP5*), *TRV2::NbXBCP26* (silencing *NbXBCP2* and *NbXBCP6*), *TRV2::NbRD19ABC* (silencing *NbRD19A*, *NbRD19B*, and *NbRD19C*), *TRV2::NbRD19E* (silencing *NbRD19E*), *TRV2::NbCTB13* (silencing *NbCTB1* and *NbCTB3*), *TRV2::NbCTB2* (silencing *NbCTB2*), and *TRV2::NbXCP123* (silencing *NbXCP1*, *NbXCP2*, and *NbXCP3*).using homologous recombination technology. Construct the *TRV2::GUS* vector as a control. Co-inject Agrobacterium containing TRV2 experimental group with TRV1 into the leaves of 2-week-old Nicotiana benthamiana as the experimental treatment, and co-inject *TRV2::GUS* with TRV1 as the control. Fifteen days later, inject *TRBO::GFP* Agrobacterium into the T and CK groups, take photos, and collect samples 3 days later. Extract RNA and total protein from the samples for subsequent quantitative fluorescence analysis and Western Blot analysis to detect the expression level of green fluorescent protein (GFP).

### 2.8 Protein extraction and Western Blot detection

*Nicotiana benthamiana* leaves were ground in liquid nitrogen using a mortar and pestle, followed by the addition of Tris-HCl buffer (pH 8.0), 2.5 M NaCl (pH 8.0), EDTA (pH 8.0), glycerol, 100 mM PMSF, and ddH_2_O. The samples were incubated on ice for 30 minutes and then centrifuged at 12,000 rpm for 10 minutes at 4°C. The supernatant was collected and mixed with 5× SDS-PAGE protein loading buffer (Solarbio P1040) at an appropriate ratio. The mixture was heated at 95°C for 10 minutes in a metal bath. Proteins were separated by 12.5% SDS-PAGE gel electrophoresis and transferred to a PVDF membrane using the Trans-Blot Turbo system (Bio-Rad, Hercules, CA). The membrane was blocked in TBS-T containing 5% non-fat milk and incubated with Anti-GFP antibody (1:8000; Abcam, ab6556) and goat anti-rabbit IgG H&amp;L (HRP) secondary antibody (1:4000; Abcam, ab6721).

### 2.9 RNA extraction and quantitative real-time PCR analysis

Whole RNA of *N. benthamiana* from control and processing group extracted with an RNA extraction kit (Beijing Kangwei Century Company, CW0581M). The cDNA was reverse transcription kit (Nanjing Norzan Biotechnology Co., Ltd., R333-01). For real-time PCR (Real-time PCR) detection, 10-fold diluted cDNA was used as the reaction template and a set of specific PCR reaction process: at the starting stage, 3 minutes pre-denaturation at 95°C; then 40 PCR cycles with 10 seconds 95°C denaturation phase and 30 seconds 60°C extension phase; and the flow parameters were 15 seconds 95°C treatment, 60 seconds 60°C treatment, and finally 15 seconds 95°C treatment. Data were calculated and analyzed using the 2^-ΔΔCT^ method (Rao et al., 2013), with *NbActin* as the reference gene. The results were compared and integrated with other data. Significance analysis was performed using the ANOVA method with a significance level set at 0.05. The specific primers applied in this trial are detailed in **Supplementary Table S2**, and three experimental replicates were performed for each sample.

## 3 Results

### 3.1 Identification and annotation of NbPLCPs

Using the HMMER search technique, a total of 50 PLCP family genes were successfully identified (**Table 1**). The corresponding proteins were analyzed using protein analysis tools to determine their physicochemical properties. The protein lengths ranged from 156 to 925 amino acids, with molecular weights ranging from 17,147.41 Da to 104,611.01 Da. The theoretical isoelectric points (pI) varied significantly, with NbRD19G showing the highest pI and NbTHI2 showing the lowest. NbXBCP6 exhibited the highest instability index, while NbRD19C had the lowest aliphatic index. These parameters enhanced our understanding of the characteristics of the NbPLCP family, providing deeper insights into their structure and physicochemical properties. Additionally, the subcellular localization of the proteins encoded by NbPLCP was predicted, and the results indicated that most family members are localized to vacuoles and the endoplasmic reticulum. Specifically, NbXBCP6, NbRD19C, and NbRD19F were predicted to be localized to the nucleus, NbRD19H was predicted to be localized to the Golgi apparatus, and NbPAP3 was predicted to be localized to the membrane, while NbCTB1 was predicted to be localized to both vacuoles and chloroplasts.

**Table 1:** Physicochemical characterization of PLCP gene family members in *Nicotiana benthamiana*.

Group	Gene ID	Gene name	Number of amino acid	Molecular weight	Theoretical pI	Instability index	Aliphatic index	Gravy	Subcellular localization
RD21	*Nbe01g03680.1*	*NbRD21A*	240	27431.64	5.33	31.13	67.04	-0.59	Vacuole
	*Nbe07g24350.1*	*NbRD21B*	470	51539.07	5.58	30.16	67.43	-0.45	Vacuole
	*Nbe12g22210.1*	*NbRD21C*	465	51108.34	5.36	36.49	69.20	-0.4	Vacuole
	*Nbe13g10990.1*	*NbRD21D*	473	51786.26	5.76	30.09	65.54	-0.48	Vacuole
	*Nbe15g02990.1*	*NbRD21E*	465	51071.20	5.12	40.99	70.02	-0.39	Vacuole
	*Nbe03g10470.1*	*NbRDL1*	374	42365.55	7.10	35.17	68.77	-0.59	Vacuole
	*Nbe04g29160.1*	*NbRDL2*	374	42280.54	6.73	36.97	70.61	-0.54	Vacuole
CEP	*Nbe05g24660.1*	*NbCEP1*	361	40508.59	6.20	35.65	66.15	-0.6	Endoplasmic reticulum
	*Nbe06g22830.1*	*NbCEP2*	361	40558.46	5.78	36.28	65.60	-0.61	Endoplasmic reticulum
	*Nbe13g08980.1*	*NbCEP3*	357	39787.79	5.64	32.33	70.73	-0.47	Endoplasmic reticulum
	*Nbe15g06380.1*	*NbCEP4*	362	40565.54	6.04	29.74	67.07	-0.6	Endoplasmic reticulum
	*Nbe16g15630.1*	*NbCEP5*	362	40452.30	5.97	29.03	67.60	-0.59	Endoplasmic reticulum
	*Nbe19g14710.1*	*NbCEP6*	357	39762.79	5.91	35.26	68.54	-0.49	Endoplasmic reticulum
XCP	*Nbe07g17300.1*	*NbXCP1*	355	39907.19	5.64	26.23	78.54	-0.39	Vacuole
	*Nbe12g34660.1*	*NbXCP2*	355	39921.29	5.74	23.05	79.92	-0.36	Vacuole
	*Nbe16g13040.1*	*NbXCP3*	353	39725.80	5.39	39.50	73.48	-0.39	Vacuole
XBCP	*Nbe03g28930.1*	*NbXBCP1*	505	56728.90	5.31	48.98	63.15	-0.53	Vacuole
	*Nbe03g28940.1*	*NbXBCP2*	503	56274.89	5.26	50.14	72.68	-0.37	Vacuole
	*Nbe07g20010.1*	*NbXBCP3*	439	48169.12	5.38	39.46	71.03	-0.31	Vacuole
	*Nbe13g14250.1*	*NbXBCP4*	438	47859.87	5.59	39.33	70.30	-0.29	Vacuole
	*Nbe17g00220.1*	*NbXBCP5*	405	44255.53	5.73	41.07	64.72	-0.41	Vacuole
	*Nbe18g28970.1*	*NbXBCP6*	615	66994.26	5.39	80.87	59.90	-0.59	Nucleus/Vacuole
	*Nbe18g28980.1*	*NbXBCP7*	501	56188.28	5.36	47.34	65.21	-0.52	Vacuole
THI	*Nbe17g07020.1*	*NbTHI1*	205	22224.09	5.78	25.21	67.56	-0.36	Endoplasmic reticulum
	*Nbe18g28670.1*	*NbTHI2*	188	19998.55	4.57	24.15	88.14	0.09	Vacuole
	*Nbe19g03320.1*	*NbTHI3*	206	22295.99	4.99	23.12	67.72	-0.34	Endoplasmic reticulum
SAG12	*Nbe03g29120.1*	*NbSAG12A*	340	37474.88	5.06	23.13	67.47	-0.38	Vacuole
	*Nbe16g10350.1*	*NbSAG12B*	332	36623.07	5.29	17.51	67.38	-0.37	Vacuole
	*Nbe17g18710.1*	*NbSAG12C*	339	38061.05	6.55	14.76	72.21	-0.38	Vacuole
	*Nbe18g19100.1*	*NbSAG12D*	340	37952.64	6.67	24.11	63.71	-0.51	Vacuole
	*Nbe18g28710.1*	*NbSAG12E*	340	37140.68	6.61	20.97	65.76	-0.38	Endoplasmic reticulum
	*Nbe18g28720.1*	*NbSAG12F*	340	37129.60	5.76	19.56	66.62	-0.37	Endoplasmic reticulum
	*Nbe18g28730.1*	*NbSAG12G*	286	31339.30	6.60	13.45	70.66	-0.29	Vacuole
	*Nbe18g28820.1*	*NbSAG12H*	289	31816.93	6.89	25.41	65.16	-0.43	Vacuole
	*Nbe17g18720.1*	*NbPAP1*	324	36210.83	5.22	36.60	79.51	-0.37	Vacuole
	*Nbe18g28680.1*	*NbPAP2*	348	38704.14	4.96	29.26	72.59	-0.36	Vacuole
	*Nbe18g28700.1*	*NbPAP3*	201	22575.73	5.96	39.02	69.45	-0.31	Cell membrane/Vacuole
RD19	*Nbe01g01530.1*	*NbRD19A*	370	40804.10	6.01	32.56	78.54	-0.29	Vacuole
	*Nbe02g13940.1*	*NbRD19B*	370	40808.02	5.95	32.17	77.46	-0.28	Vacuole
	*Nbe05g01930.1*	*NbRD19C*	925	104611.01	8.43	50.63	55.63	-0.98	Nucleus
	*Nbe06g32440.1*	*NbRD19D*	387	42571.46	6.73	31.08	77.65	-0.21	Vacuole
	*Nbe07g19880.1*	*NbRD19E*	365	40284.39	6.13	29.43	75.95	-0.33	Vacuole
	*Nbe08g09060.1*	*NbRD19F*	156	17147.41	8.25	52.12	70.71	-0.4	Nucleus/Vacuole
	*Nbe12g02110.1*	*NbRD19G*	265	30432.11	9.01	31.97	79.89	-0.42	Vacuole
	*Nbe14g32420.1*	*NbRD19H*	238	26538.79	6.00	38.60	68.11	-0.57	Golgi/Vacuole
ALP	*Nbe09g10400.1*	*NbALP1*	359	39666.02	8.25	37.56	79.86	-0.25	Vacuole
	*Nbe14g17540.1*	*NbALP2*	360	39207.60	6.94	23.55	81.28	-0.13	Vacuole
CTB	*Nbe03g25810.1*	*NbCTB1*	862	95608.36	5.11	40.99	94.33	-0.11	Chloroplast/Vacuole
	*Nbe03g25840.1*	*NbCTB2*	357	39586.01	6.12	37.47	80.03	-0.22	Vacuole
	*Nbe04g13100.1*	*NbCTB3*	361	39876.46	6.00	37.69	83.77	-0.16	Vacuole

### 3.2 Phyloevolutionary analysis of the *NbPLCP* families

**Figure 1:**
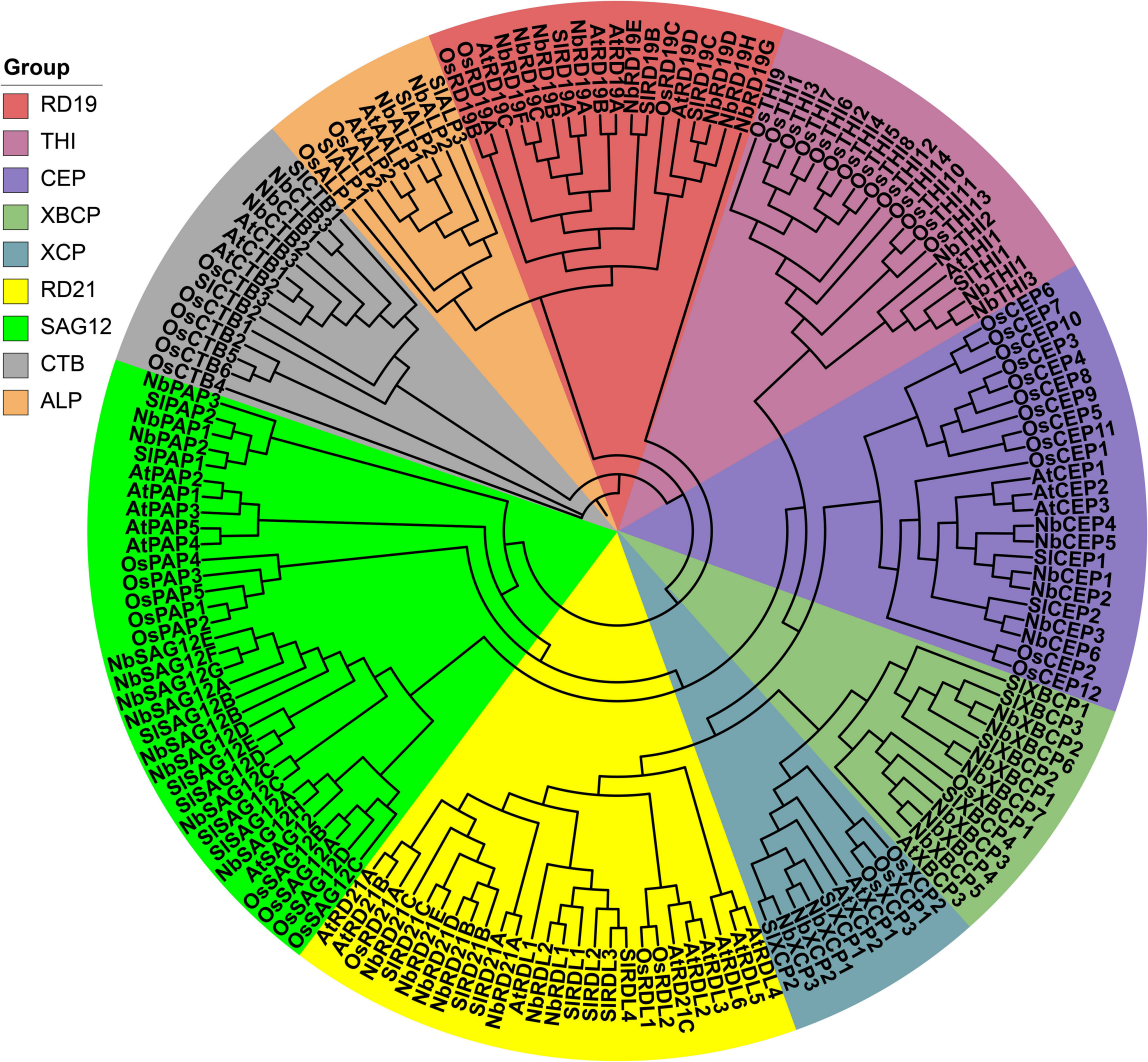
Evolutionary relationships of members of the NbPLCP family with *Arabidopsis*, tomato and rice.

To further investigate the evolutionary relationships of *NbPLCP*, we constructed a phylogenetic tree using PLCP protein sequences from different species (**Figure 1**). Based on previous studies, PLCPs were classified into nine subfamilies: CTB, ALP, RD19, SAG12, CEP, THI, XBCP, XCP, and RD21. The *NbPLCP* family members are distributed across these nine subfamilies. The ALP subfamily contains the fewest *NbPLCP* members, with only two members; the CTB, THI, and XCP subfamilies each contain three members; the CEP subfamily has six members; the RD21 and XBCP subfamilies each have seven members; and the RD19 subfamily contains eight members. The SAG12 subfamily has the largest number of *NbPLCP* members, with eleven. Evolutionary analysis across different species indicates that the evolutionary mechanisms of the PLCP gene family are relatively conserved.

### 3.3 Conserved motifs, protein structure, and gene structure analysis of *NbPLCPs*

**Figure 2:**
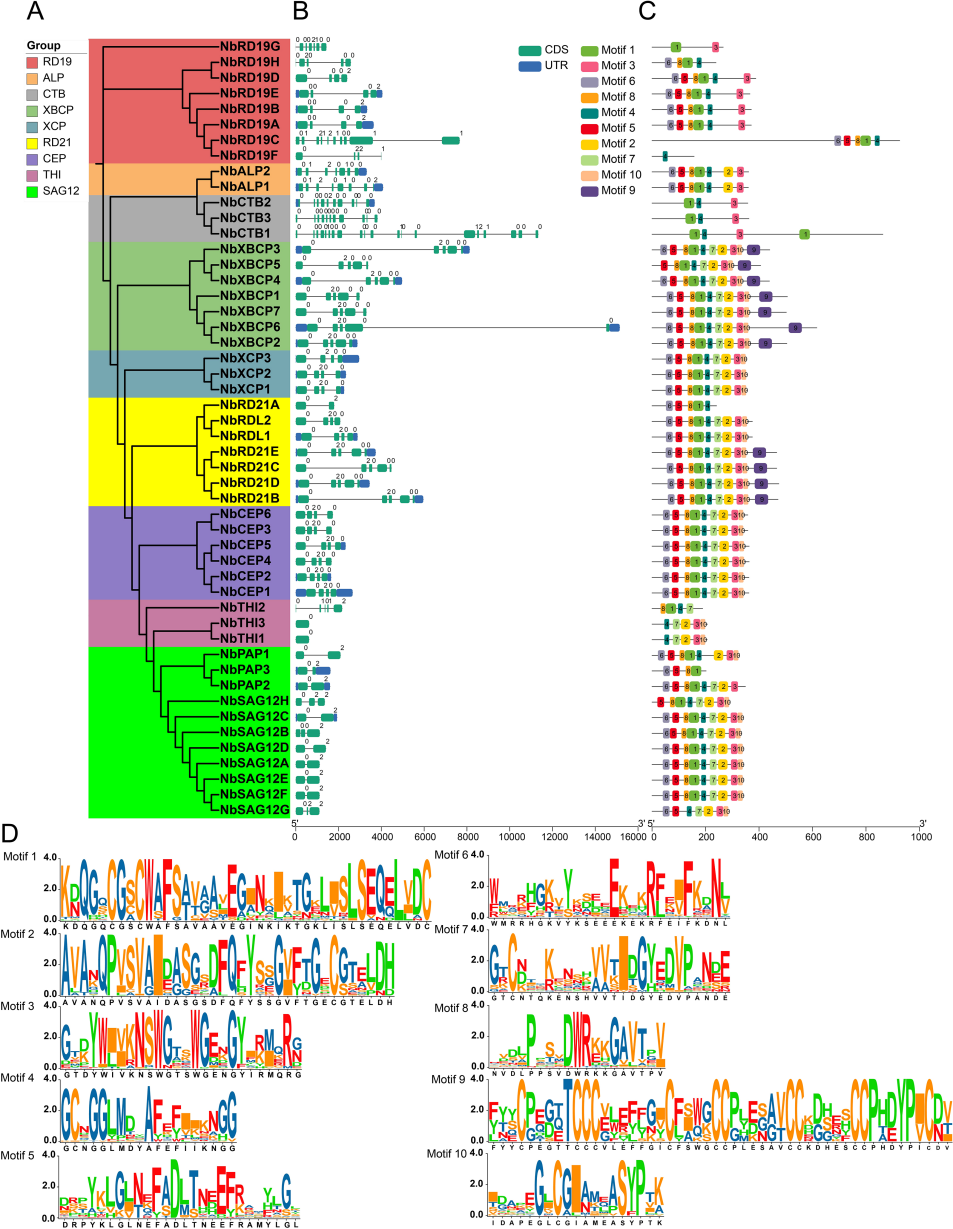
Structure and motif analysis of the *NbPLCPs* gene. **(A)** Phylogenetic tree of *NbPLCPs* constructed using MEGA. **(B)** A map of the exon structures displayed using the GSDS web site. **(C)** Analysis of 10 Motifs in the meme website construction. **(D)** Analysis of the conserved motifs.

We analyzed the gene structure of *NbPLCPs*, and showed that these genes contained more than one exon, ranging from 1 to 20 (**Figure 2**), number of the same exons for the same members of the same subfamily.Such as the number of exons in the XCP subfamily, the XBCP subfamily, and the CEP subfamily. The member with the highest number of exons, *NbCTB1*, located in the CTB subfamily, has 20 exons. Although other subfamily members do not have uniform numbers of exons, they possess very close numbers of exons.

To further understand the structural and functional characteristics of NbPLCPs family members, 10 conserved motifs in NbPLCPs proteins were identified using MEME software (**Figure 2**), with only one Motif4 in NbRD19F and all members except NbRD19G without Motif4 having Motif4. Where Motif1 is present in other members except the four members, NbRD19F, NbTHI1, NbTHI3 and NbSAG12G. Combined with the phylogenetic tree, we shows that the protein structure of the members on the same branch is basically consistent, suggesting that the members on the same branch may continue their function. In summary, the analysis of conserved motifs, protein domains, and gene structure of gene family members provides strong evidence for the results of the phylogenetic analysis.

### 3.4 Gene duplication analysis and chromosomal distribution of *NbPLCPs*

**Figure 3:**
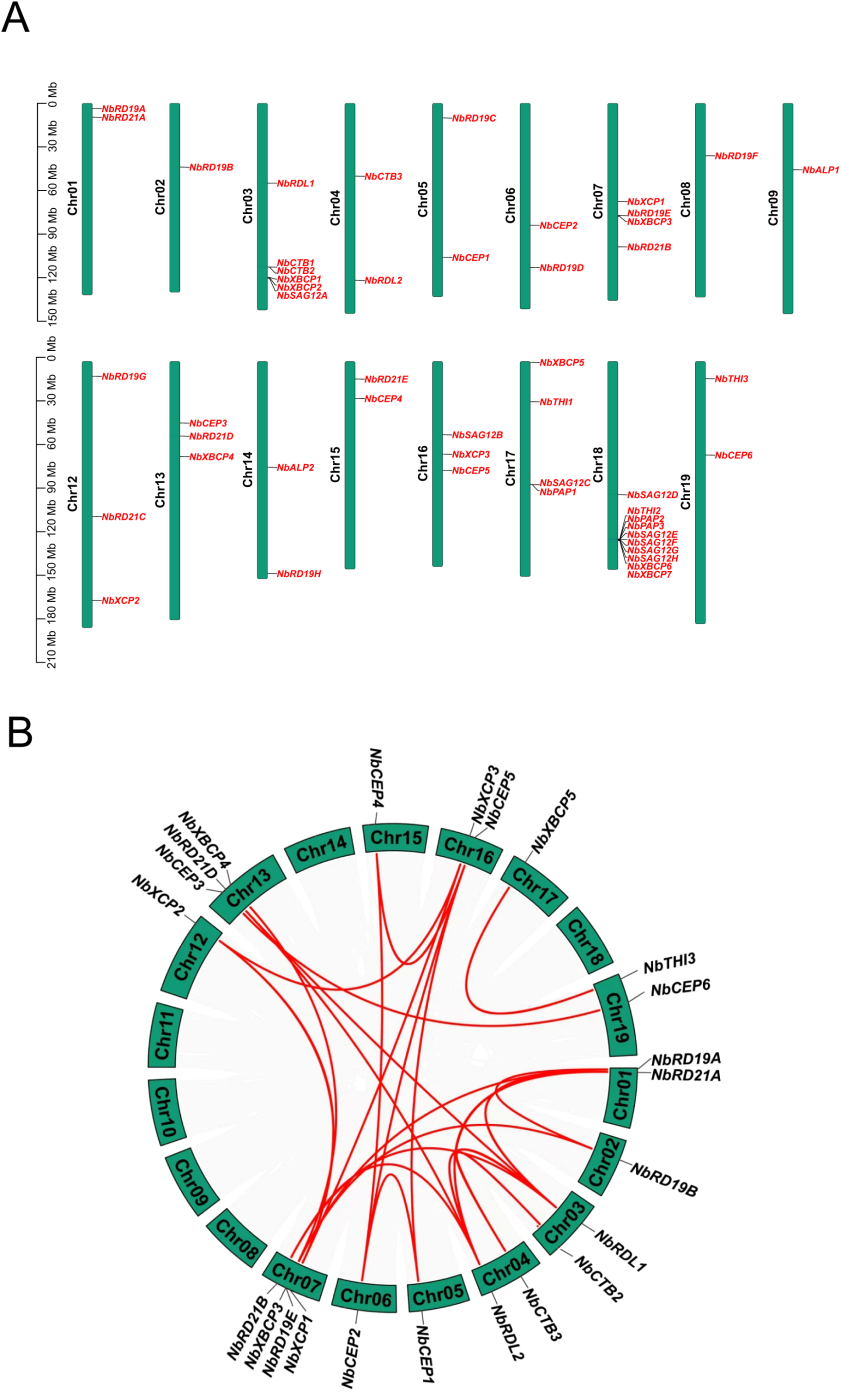
**(A)** Chromosome positioning is based on the location of 17 chromosomes (Mb), the proportion on the left is trillion 1 billion (Mb), and the number of chromosomes is located on the left of each chromosome. **(B)** The inter-genomic collinearity of PLCP genes in *N. benthamiana*. The red line represents the segmental duplication event among PLCP family members.

In order to better understand the distribution of the *NbPLCP* gene family members across the genome, based on the current *N. benthamiana* genome, 50 *NbPLCP* genes are unevenly distributed across 17 of the 19 chromosomes (**Figure 3**). Chromosome 18 harbors the highest number of *NbPLCP* genes (10 genes), while chromosomes 2, 8, and 9 contain only one *NbPLCP* gene each. Two *NbPLCP* genes are located on chromosomes 1, 4, 5, 6, 14, 15, and 19 respectively, whereas three *NbPLCP* genes are found on chromosomes 12, 13, and 16. Chromosomes 7 and 17 each possess four *NbPLCP* genes, and chromosome 3 accommodates six *NbPLCP* genes. Subsequent analyses identified 22 segmental duplication events among 23 *NbPLCP* genes, with the *NbXBCP5/NbTHI3* segmental duplication occurring across two subfamilies, while the remaining 21 segmental duplications occurred within the same subfamily. Notably, no tandem duplications were detected, suggesting that segmental duplications play a pivotal role in the evolution of the *NbPLCP* gene family. Additionally, the selection pressure analysis of the *NbPLCP* genes (**Table 2**) revealed that the Ka/Ks value for the *NbXBCP5/NbTHI3* gene pair (0.543), although less than 1, is greater than 0.5, indicating strong functional constraints on these genes during evolution, with certain sites undergoing adaptive changes.

**Table 2:** Ratios of nonsynonymous (Ka) and synonymous (Ks) of *NbPLCP* gene fragment duplication pairs in *Nicotiana benthamiana*.

Gene pairs	Ka	Ks	Ka/Ks
*NbRD19A/NbRD19B*	0.014	0.148	0.096
*NbRD19A/NbRD19E*	0.195	1.659	0.118
*NbRD21A/NbRDL1*	0.285	3.781	0.075
*NbRD21A/NbRDL2*	0.312	2.760	0.113
*NbRD19B/NbRD19E*	0.213	2.023	0.105
*NbRDL1/NbRDL2*	0.027	0.139	0.193
*NbRDL1/NbRD21B*	0.337	1.594	0.212
*NbRDL1/NbRD21D*	0.331	1.613	0.205
*NbCTB2/NbCTB3*	0.127	0.450	0.281
*NbRDL2/NbRD21B*	0.334	1.910	0.175
*NbRDL2/NbRD21D*	0.341	1.774	0.192
*NbCEP1/NbCEP2*	0.008	0.145	0.057
*NbCEP1/NbCEP5*	0.086	0.627	0.137
*NbCEP2/NbCEP4*	0.074	0.640	0.116
*NbCEP2/NbCEP5*	0.079	0.610	0.130
*NbXCP1/NbXCP2*	0.011	0.156	0.069
*NbXCP1/NbXCP3*	0.081	0.770	0.106
*NbXBCP3/NbXBCP4*	0.020	0.108	0.184
*NbXCP2/NbXCP3*	0.082	0.663	0.124
*NbCEP3/NbCEP6*	0.013	0.086	0.154
*NbCEP4/NbCEP5*	0.014	0.118	0.122
*NbXBCP5/NbTHI3*	0.027	0.049	0.543

### 3.5 Investigation of co-linear relationships between *N. benthamiana* and other species

**Figure 4:**
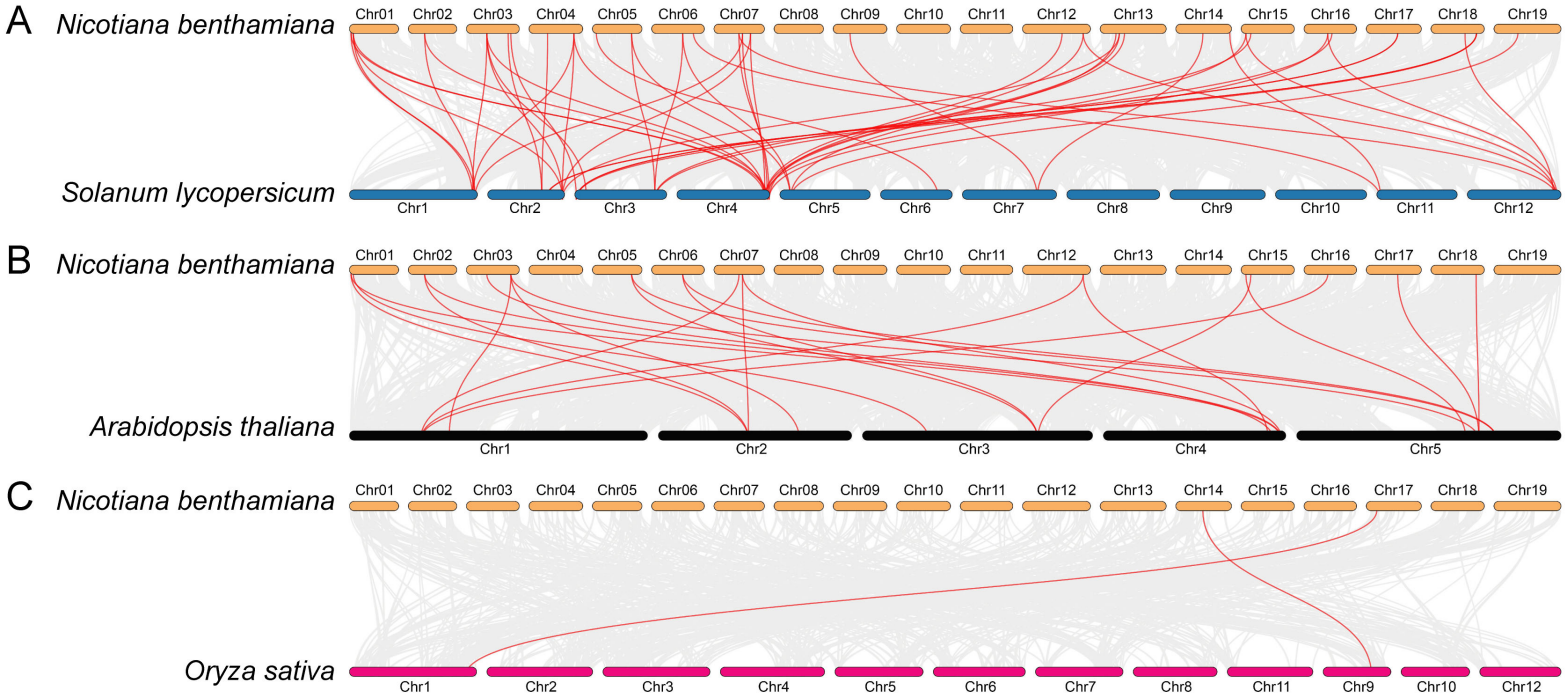
Synteny analysis between *Nicotiana benthamiana* and other species. Gray lines indicate all collinear relationships between different chromosomes, while colored lines represent collinear analysis specific to *NbPLCP* family genes. **(A)** Synteny analysis between *N. benthamiana* and *Solanum lycopersicum*. **(B)** Synteny analysis between *N. benthamiana* and *Arabidopsis thaliana*. **(C)** Synteny analysis between *N. benthamiana* and *Oryza sativa*.

In order to enhance our understanding of the evolutionary relationships within the *NbPLCP* gene family, we conducted a comparative analysis. This analysis examined the collinearity of *NbPLCP* genes in *N. benthamiana* with those from three other plant species (see **Figure 4**). The results indicated that there are 33, 15, and 2 *NbPLCP* genes exhibiting collinearity with the *NbPLCP* genes of tomato, Arabidopsis, and rice, respectively. Among these three species, the number of collinear gene pairs between *N. benthamiana* and each species was found to be 52, 23, and 2, respectively. Notably, the genes *NbRD19A, NbRD21A, NbRDL1, NbSAG12A, NbRDL2, NbCEP1*, and *NbXCP2* in *N. benthamiana* displayed consistent chromosomal positions with their homologous genes in tomato, suggesting that these genes have maintained a similar genomic structure throughout evolution. In contrast, within the collinear gene pairs with *Arabidopsis*, only *NbCEP1* exhibited consistent chromosomal positioning with its homologous gene in *Arabidopsis*. The differences in ecological adaptability between *N. benthamiana* and rice reflect the distinct selective pressures they have encountered during evolution, leading to diversity in genomic structure and function. The comparative genomic data between *N. benthamiana*, tomato, *Arabidopsis*, and rice reveal significant insights into their evolutionary relationships, gene homology, and functional conservation, providing a foundational understanding of plant genetic diversity and evolutionary history.

### 3.6 Analysis of promoter cis-acting elements of *NbPLCP* gene

**Figure 5:**
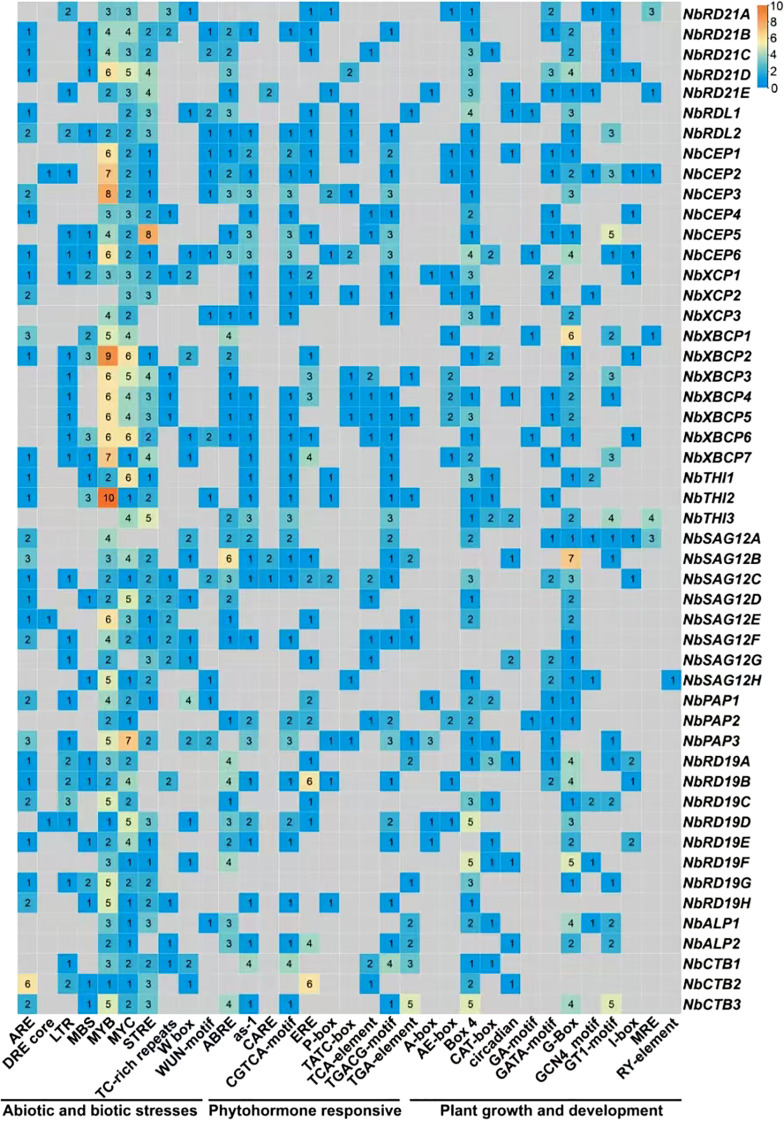
Analysis of promoter cis-acting elements in response to light signal, phytohormone, growth and development, and abiotic stress.

Research into *NbPLCP*’s potential involvement in stress responses necessitates a thorough analysis of the cis-acting elements within the promoter regions of these genes. This study aims to uncover various elements associated with abiotic and biotic stresses, plant hormones, and growth and development (**Figure 5**), offering significant insights into the regulatory mechanisms of *NbPLCP* in response to environmental stimuli. Under abiotic and biotic stresses, genes in the *NbRD21* subfamily display diverse expression patterns under adverse conditions. Notably, *NbRD21D* possesses high binding capacities to promoter elements like DRE and MBS, highlighting its crucial role in drought and salt stresses. By modulating the expression of genes related to stress resistance, members of the *NbRD21* subfamily enhance the plant’s adaptability to environmental pressures. *NbCEP2* shows maximum enrichment under cold and salt stresses, indicating its significance in adversity response. Moreover, *NbXCP1*’s high enrichment under oxidative stress suggests its link to plant antioxidative capabilities, potentially enhancing stress resistance through the modulation of relevant signaling pathways. Within the *NbSAG12* subfamily, increased enrichment of *NbSAG12B* on drought and high temperature regulatory elements suggests its key role in plant senescence and stress response. Additionally, changes in the enrichment of adversity cis-acting elements in members of the *NbALP* and *NbCTB* subfamilies also demonstrate their roles in plant growth and stress resistance. Particularly, the cold and saline-alkali stress elements of *NbCTB2* underscore its critical role in plant stress resistance. Finally, variations in multiple adversity elements in members of the *NbRD19* subfamily further emphasize the importance of promoter elements in regulating plant stress responses.

### 3.7 *NbPLCP* dynamic expression patterns in response to viral infection

**Figure 6:**
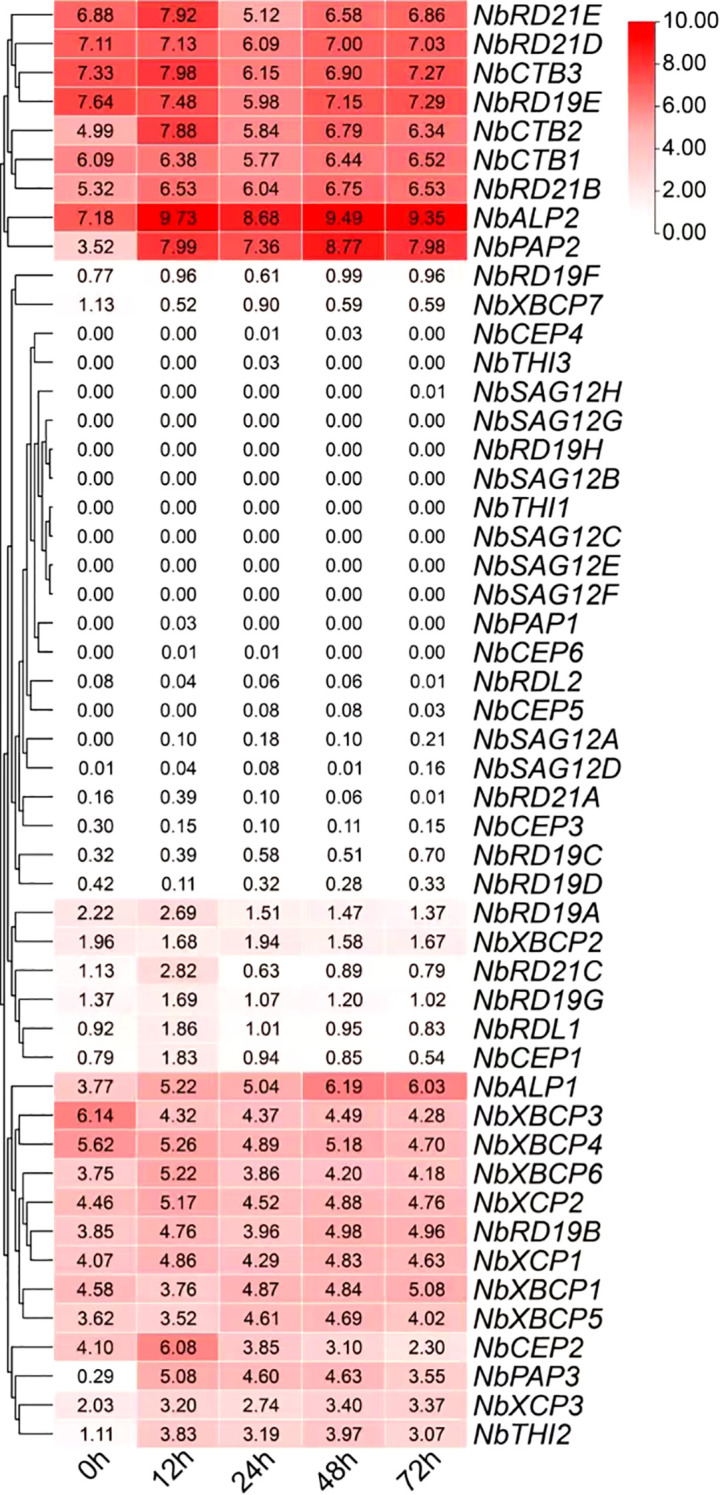
Analysis of *NbPLCP* gene expression patterns under viral infection treatment based on RNA-seq data. The legend represents the expression levels of *NbPLCP* genes, ranging from high expression (red) to low expression (white).

To gain a better understanding of the role of the *NbPLCP* protease family in the *N. benthamiana* bioreactor, this study analyzed the dynamic expression patterns of *NbPLCP* in response to viral infection (**Figure 6**). Most members of this family responded to viral infection at varying degrees across different time points, with diverse time-dependent expression characteristics observed among different genes during the infection process, reflecting their potential roles in plant immune responses. Furthermore, some genes displayed stage-specific expression peaks, such as *NbRD21C*, which significantly increased after 12 hours of infection and then decreased, suggesting that it may have a key regulatory function at specific stages of infection. Similarly, the *NbXCP* and *NbRD19* subfamily genes exhibited coordinated expression changes, indicating that they may be regulated by a common regulatory network, potentially participating in the regulation of the same biological pathway or engaging in crosstalk between different immune signaling pathways. The synchronous expression observed within gene clusters further suggests the involvement of potential regulatory factors and co-regulatory mechanisms in driving their responses. For instance, *NbXCP1*, *NbXCP2*, and *NbXCP3* exhibited similar expression patterns, gradually increasing after infection, peaking at 48 hours, and then slightly declining thereafter. These findings highlight the intricate regulatory networks and diverse functional roles of *NbPLCP* family members in plant immunity.

### 3.8 The protease inhibitor SICYS8 enhances the expression of exogenous GFP protein

**Figure 7:**
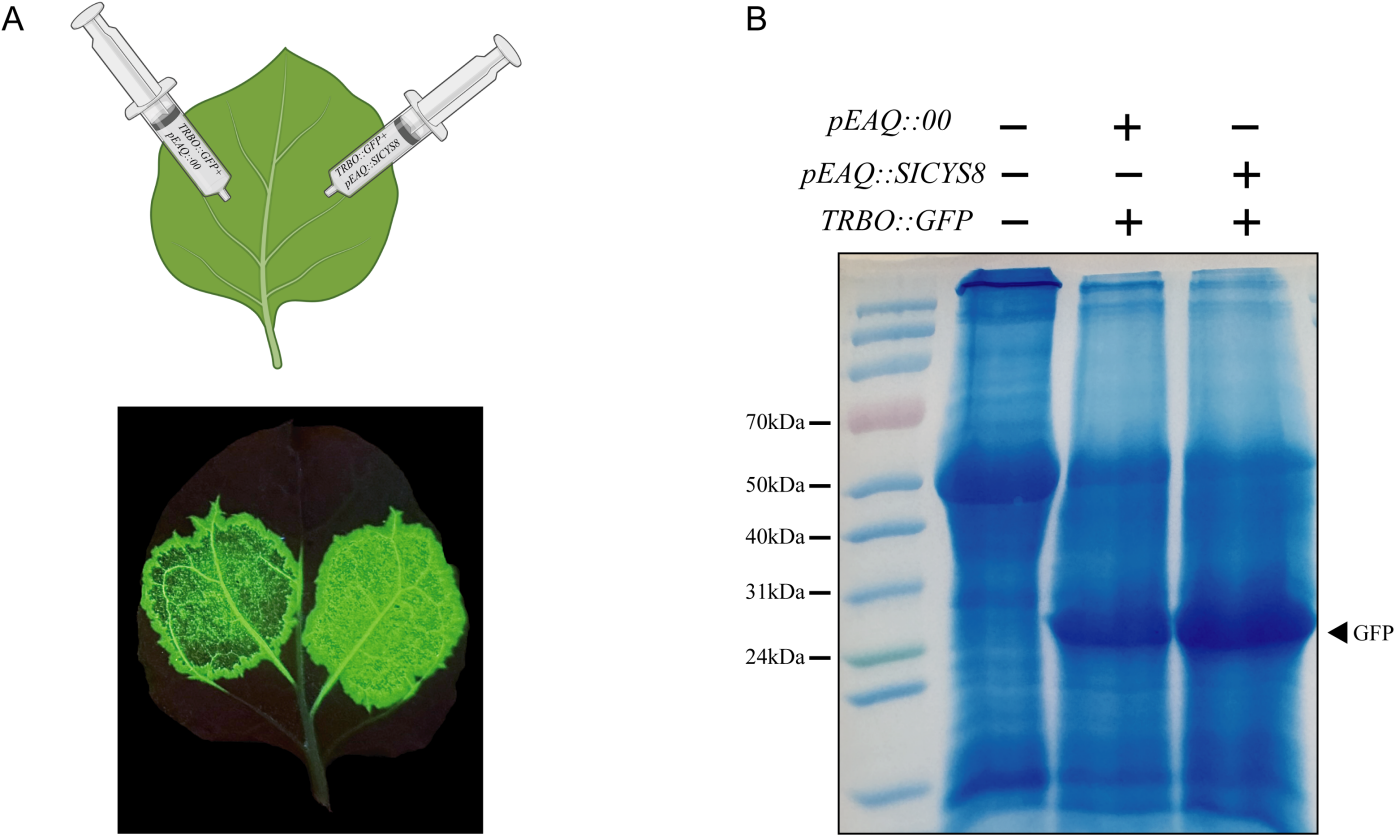
**(A)** Agroinfiltration of *Nicotiana benthamiana* leaves, with 35s::GFP infiltrated on the left side and a 1:1 mixture of 35s::GFP and 35s::SICYS8 infiltrated on the right side. **(B)** Western blot analysis of the samples from panel B, showing the detection results of GFP protein (molecular weight: 27 kDa).

Previous studies have shown that the protease inhibitor SICYS8 inhibits the hydrolytic activity of the PLCP protease family, thereby increasing the expression of exogenous proteins in *N. benthamiana*. In this study, we transiently expressed GFP along with SICYS8 in *N. benthamiana* to investigate the impact of the NbPLCP protease inhibitor SICYS8 on the biological response of the *N. benthamiana* bioreactor. From the phenotype, it was observed that the green fluorescence intensity on the right side, where only GFP was expressed, was lower than on the left side, where both SICYS8 and GFP were co-expressed (**Figure 7**). Western blot analysis revealed that the GFP protein content was significantly higher in the samples co-expressing SICYS8 compared to the control group (**Figure 7**). This indicates that SICYS8 can enhance the expression of recombinant proteins in *N. benthamiana* by inhibiting the activity of proteinases.

### 3.9 The effect of PLCP family members on the expression of exogenous GFP in *Nicotiana benthamiana*

As shown in **Figure 6**, following infection with exogenous viruses, changes in the expression levels of PLCP family members are primarily concentrated in the NbRD21, NbCTB, NbRD19, NbXBCP, and NbCEP subfamilies. Based on the phylogenetic relationships of these subfamily members illustrated in **Figure 2**, we constructed ten transient silencing vectors: *TRV2::NbRD21BD, TRV2::NbRD21E, TRV2::NbXBCP17, TRV2::NbXBCP345, TRV2::NbXBCP26, TRV2::NbRD19ABC, TRV2::NbRD19E, TRV2::NbCTB13, TRV2::NbCTB2*, and *TRV2::NbXCP123*. Using transient silencing technology, these genes were knocked down. Following the knockdown, the plants were infected with green fluorescent protein (GFP), and the GFP expression level was observed and measured.

**Figure 8:**
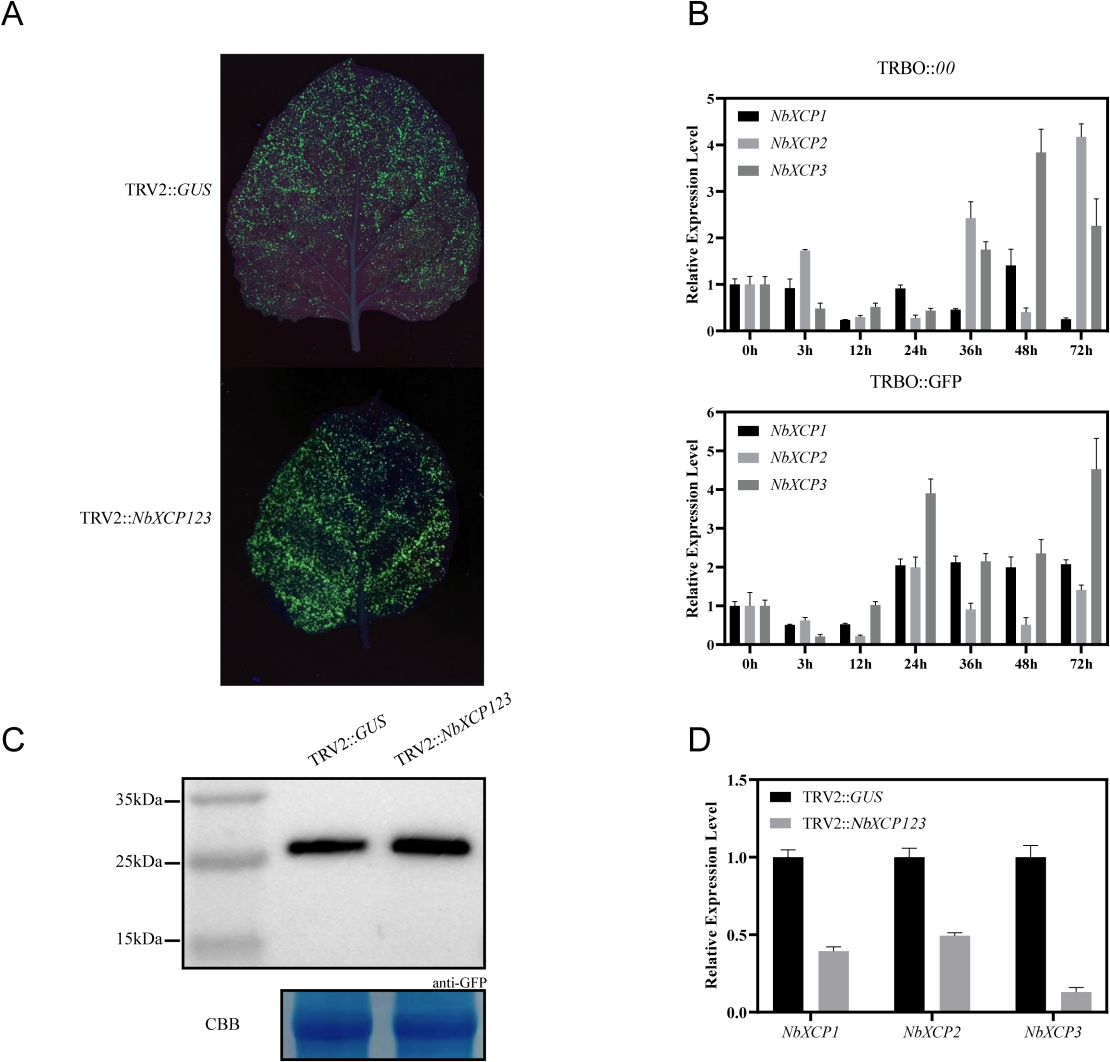
**(A)** Phenotypic comparison of *TRBO::GFP* infection in control plants and NbXCP123-silenced plants. **(B)** The expression levels of *NbXCP1*, *NbXCP2*, and *NbXCP3* in *Nicotiana benthamiana* infected with *TRBO::00* and *TRBO::GFP* were analyzed at 0 h, 3 h, 12 h, 24 h, 36 h, 48 h, and 72 (h) **(C)** Western blot analysis of GFP expression in control plants and NbXCP123-silenced plants. **(D)** Expression levels of *NbXCP1, NbXCP2*, and *NbXCP3* in control plants and NbXCP123-silenced plants.

The results of the comparison showed that GFP fluorescence intensity in NbXCP123-silenced plants was higher than that in the control plants (**Figure 8**). As shown in **Figure 8**, the expression levels of three genes, *NbXCP1*, *NbXCP2*, and *NbXCP3*, were analyzed in Nicotiana benthamiana leaves infected with either GFP or an empty vector control over a three-day period. Compared to the control group, significant changes were observed in the GFP-infected plants after 24 hours. Specifically, the expression of *NbXCP1* and *NbXCP3* increased overall and stabilized after 24 hours, while *NbXCP2* showed an initial increase followed by a decrease. **Figure 8** presents the results of a Western blot analysis of GFP expression in NbXCP123-silenced plants and control plants. The results indicate that GFP expression in the NbXCP123-silenced plants was higher than in the control plants. As shown in **Figure 8**, the three target genes, *NbXCP1*, *NbXCP2*, and *NbXCP3*, were significantly downregulated in NbXCP123-silenced plants compared to the control plants. These results demonstrate that the expression levels of XCP subfamily members in *Nicotiana benthamiana* were indeed altered after the infection with the exogenous GFP protein, showing a marked increase. Moreover, an obvious elevation in GFP expression was observed and detected in the transiently silenced plants.

## 4 Discussion

The PLCP (Papain-Like Cysteine Protease) family is one of the most abundant cysteine protease families in plants, participating in various life processes, including senescence, pollen development, fruit ripening, and seed germination (Liu et al., 2018). To date, systematic analyses of the PLCP family have been conducted in species such as *Arabidopsis thaliana* (Richau et al., 2012), rice (Nino et al., 2020), chili pepper (Chen et al., 2024), soybean (Yuan et al., 2020), grape (Kang et al., 2021), and papaya (Liu et al., 2018). In this study, we identified 50 members of the PLCP family in the genome of *N. benthamiana*. Phylogenetic analysis classified these genes into nine subfamilies, with members of the same subfamily showing similarities in gene structure and conserved motifs. The differences in amino acid length, molecular weight, isoelectric point, aliphatic amino acid ratio, hydrophobicity index, and chromosomal location of NbPLCP proteins suggest that these variations may be related to the functional diversity of these family members.

Gene structure analysis reveals significant differences in intron and exon compositions among the members of the *N. benthamiana PLCP* gene family. For example, members of the XCP, XBCP, and CEP subfamilies exhibit consistency in the number of exons, reflecting the conservation of these genes in specific biological functions. The lack of conserved motifs in some PLCP family members suggests potential functional diversification within the family, indicating that NbPLCP proteins may play diverse roles in various physiological processes. Members from the same evolutionary branch display highly consistent protein structures, pointing to functional conservation throughout evolution. By integrating conserved motif and gene structure analysis, the reliability of the phylogenetic tree is further validated, providing strong support for functional predictions of family members.

Additionally, 50 members of the *N. benthamiana* PLCP gene family are distributed across 17 chromosomes (**Table 1**, **Figure 3**). Among these, 22 pairs of segmental duplication genes were identified, involving 23 *NbPLCP* genes, while no tandem duplication gene pairs were detected. This contrasts with previous studies in other species, where tandem duplication gene pairs were more likely to occur than segmental duplications within the PLCP family members of grapevines (Kang et al., 2021). Similar findings were also observed in papaya (Liu et al., 2018). This suggests that segmental duplication plays a crucial role in the evolution of the *NbPLCP* gene family. Moreover, the Ka/Ks ratio for all gene pairs was less than 1, indicating that these gene pairs have undergone purifying selection to prevent the spread of harmful mutations. Comparative genomics analysis revealed 52 pairs of collinear genes between *N. benthamiana* and tomato, 23 pairs with *Arabidopsis*, and 2 pairs with rice. These results highlight the significant evolutionary relationship, gene homology, and functional conservation between *N. benthamiana* and these species, providing fundamental insights into plant genetic diversity and evolutionary history.

This study provides an in-depth analysis of the phylogeny, gene structure, and the impact of *PLCP* genes on the expression of exogenous green fluorescent protein (GFP), revealing the importance of *PLCPs* in plant physiological processes and their potential applications in bioreactors. Previous studies have shown that PLCP protease families are common targets for pathogen effectors (Misas-Villamil et al., 2016), such as *XCP2* (Zhang et al., 2014) and *CEP2* (Mueller et al., 2013). The *VvRD21–1* gene plays a significant role in disease resistance in *Vitis vinifera L* (Kang et al., 2021). Similarly, in cotton, the orthologous gene *GhRD21–7* of *NbRD21D* in *N. benthamiana* has been shown to enhance resistance to *Verticillium dahliae* in overexpression plants (Zhang et al., 2019). The results indicate that PLCP family members exhibit significant expression changes during plant development and stress responses. We found that genes such as *NbTHI2* and *NbCEP1* are rapidly activated in the early stages of infection, exhibiting characteristics typical of acute response mechanisms, suggesting their involvement in the initiation of defense responses and signal transduction during the early stages of viral invasion. In contrast, genes such as *NbRD21D* and *NbALP2* maintain high expression levels throughout the infection process, suggesting they may play broader regulatory roles at multiple stages of the immune response. These findings suggest that silencing, knockout, or mutation of PLCP family members in plants makes them more susceptible to pathogen infection.

Through functional analysis of *PLCP* family members, we found that specific *PLCP* inhibitors (such as SICYS8) can significantly enhance the expression of exogenous GFP, indicating that the interference with endogenous proteases is a key factor affecting their accumulation. In the experiments, the GFP expression in plants with NbXCP123 silencing was significantly higher than in the control group, further validating the potential role of PLCPs in regulating exogenous protein expression. This finding provides a new strategy for optimizing N. benthamiana as a bioreactor, where the silencing or knockout of the genes *NbXCP1*, *NbXCP2*, and *NbXCP3* can potentially enhance the yield of vaccines and therapeutic proteins. Furthermore, the phylogenetic and gene structure analysis of the PLCP family lays the foundation for understanding its functional diversity. Although some PLCP members have been studied previously, this research fills the gap in phylogenetic analysis, revealing the conservation and specificity of PLCPs in different plant species. This work paves the way for future comparative functional studies of PLCPs in multiple plant species.

## 5 Conclusion

In summary, this study identified 50 members of the NbPLCP protease family through genome-wide analysis. The functional roles of the PLCP family in *N . benthamiana* and their importance in exogenous protein expression were thoroughly investigated, highlighting the potential of PLCP in plant physiology and biotechnological applications. Future research should further explore the regulatory mechanisms of PLCP and how these mechanisms can be leveraged to improve plant stress resistance and the efficiency of exogenous protein expression, thereby advancing the application of plant-based bioreactors.

## Data Availability

The datasets presented in this study can be found in online repositories. The names of the repository/repositories and accession number(s) can be found in the article/**Supplementary Material**.
