# Supplementary material for: Papain-like cysteine proteases in Nicotiana benthamiana: gene family members and their potential implications in recombinant protein expression
Source: Front Plant Sci. 2025 Jun 19;16:1565487. doi: 10.3389/fpls.2025.1565487 (PMC12224012; doi:10.3389/fpls.2025.1565487)
Supplement: Supplementary file 1 [file DataSheet1.zip › Supplementary pictures.pptx]

## Slide 1
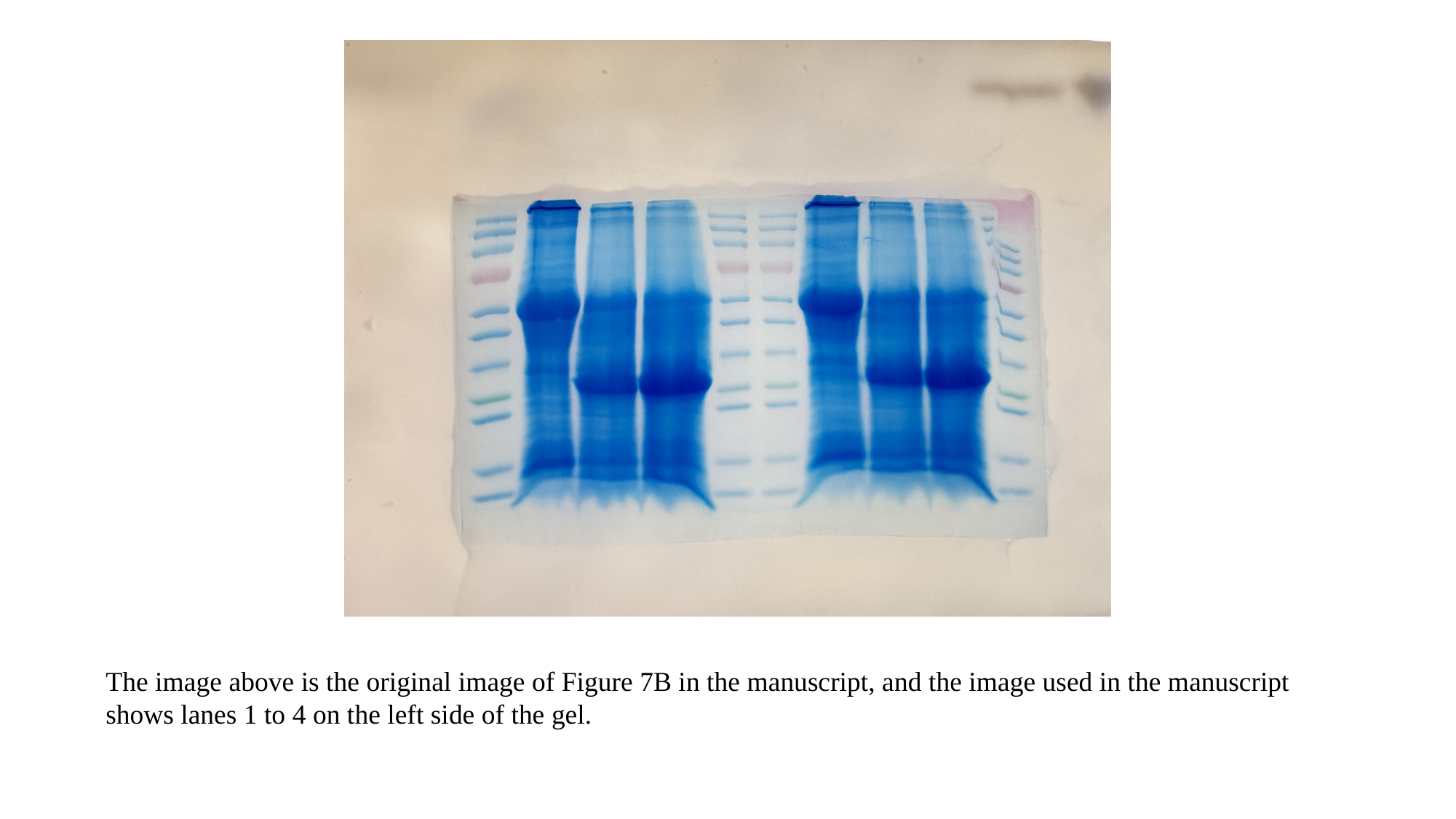

The image above is the original image of Figure 7B in the manuscript, and the image used in the manuscript shows lanes 1 to 4 on the left side of the gel.

## Slide 2
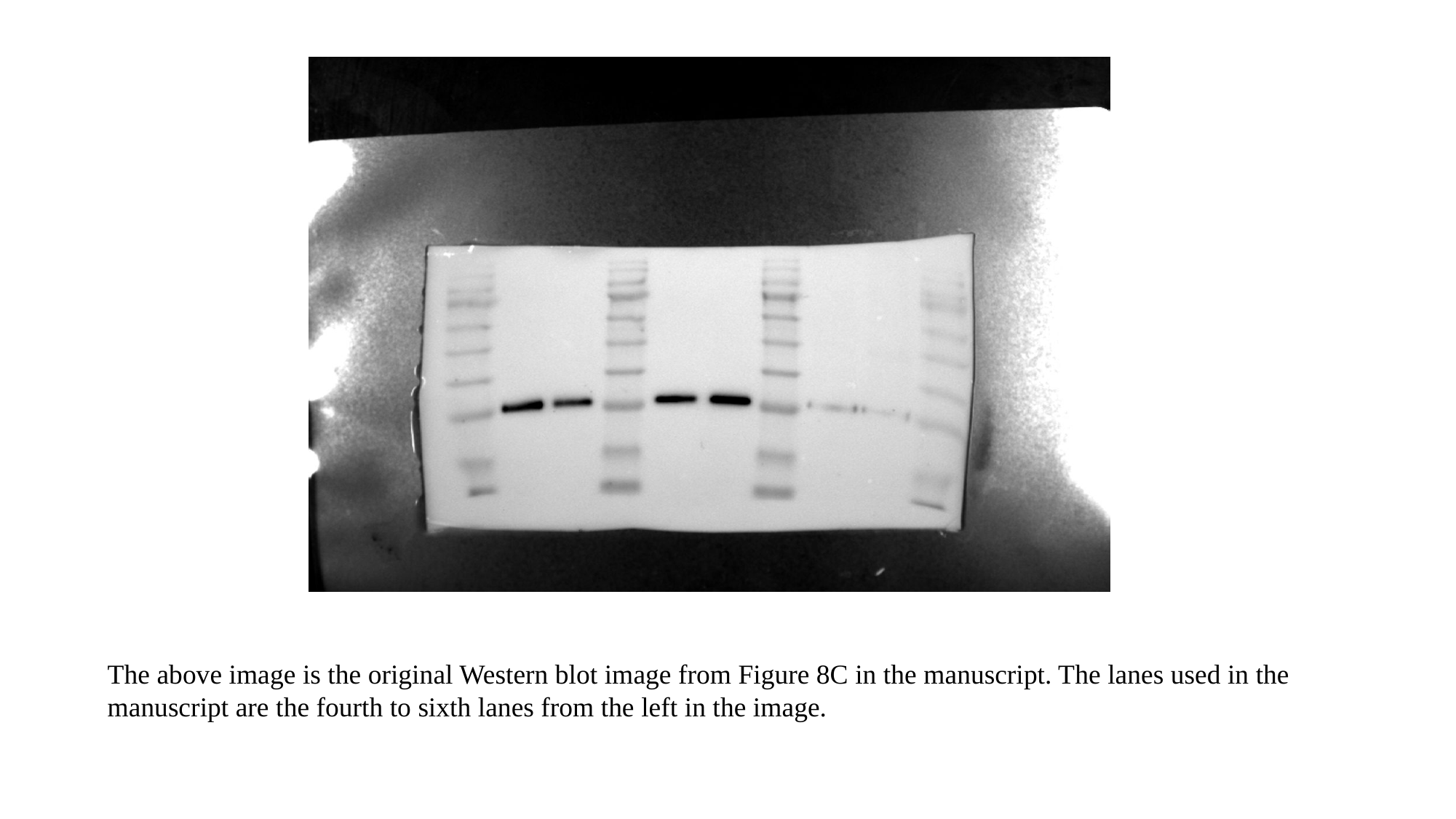

The above image is the original Western blot image from Figure 8C in the manuscript. The lanes used in the manuscript are the fourth to sixth lanes from the left in the image.
