# Supplementary material for: Papain-like cysteine proteases in Nicotiana benthamiana: gene family members and their potential implications in recombinant protein expression
Source: Front Plant Sci. 2025 Jun 19;16:1565487. doi: 10.3389/fpls.2025.1565487 (PMC12224012; doi:10.3389/fpls.2025.1565487)
Supplement: Supplementary file 2 [file Table1.docx]

Table S1: Primers used for constructing the transient silencing vector.

| Gene Name | primer F | primer R |
| --- | --- | --- |
| *NbRD21BD* | AGTAAGGTTACCGAAGCGGTTGCTTCAATT | GAGACGCGTGAGCTCACCCGCGACGGCCTT |
| *NbRD21E* | AGTAAGGTTACCGAACTACCCTTACACAGG | GAGACGCGTGAGCTCCAACGAGCACCCCAA |
| *NbXCP123* | AGTAAGGTTACCGAAGACTAATGTCAAGAA | GAGACGCGTGAGCTCCTTGAGAGACAATAA |
| *NbXBCP17* | AGTAAGGTTACCGAAGGAAAATAACTCTAA | GAGACGCGTGAGCTCAATCCTTTGATGGAG |
| *NbXBCP345* | AGTAAGGTTACCGAATGTATAGGCTCAAGG | GAGACGCGTGAGCTCTACAACTCCAGCAGA |
| *NbXBCP26* | AGTAAGGTTACCGAAGATATGAGCAATGAA | GAGACGCGTGAGCTCGCCCAGCAAGCTCCG |
| *NbRD19E* | AGTAAGGTTACCGAAACCATGAGTGTGACC | GAGACGCGTGAGCTCAGTGGCACTTGCCAT |
| *NbCTB2* | AGTAAGGTTACCGAATTTTACCGTCTACGA | GAGACGCGTGAGCTCATCGAGGCATCAAGG |
| *NbCTB13* | AGTAAGGTTACCGAACGAGGGATGTTCCCA | GAGACGCGTGAGCTCTCTCCATTCTCAGTT |
| *NbRD19ABC* | AGTAAGGTTACCGAACTAACTTGCGCCGTG | GAGACGCGTGAGCTCCAGCACGAGCCGCAT |

| Gene Name | primer F | primer R |
| --- | --- | --- |
| *NbActin* | GGTCCAGACATAGTAAGGATTGACAGA | AGACAAATCGCTCCACCAACTAAG |
| *NbXCP1* | TTGGAGAAAGAAAGGTGCTGT | CTACGGTTGAGAACGCCCA |
| *NbXCP2* | ACACTCTATGTACGACGTAACG | CCCAGAAAACCGTATTGTAACC |
| *NbXCP3* | CAGTAGCGGCAGTAGAGGGA | CTCCTTGTGAAGACCACCTTGA |

Table S2: Primers used for qRT-PCR.
